# Activity of octyl gallate against drug-sensitive and buparvaquone-resistant *Theileria annulata*

**DOI:** 10.1016/j.ijpddr.2026.100657

**Published:** 2026-07-09

**Authors:** Jin Che, Yixuan Wu, Yikang Chen, Jinming Wang, Qingli Niu, Wei Li, Shuai Yang Zhao, Guiquan Guan, Hong Yin

**Affiliations:** a State Key Laboratory of Animal Disease Control and Prevention, Key Laboratory of Veterinary Parasitology of Gansu Province, Lanzhou Veterinary Research Institute, Chinese Academy of Agricultural Sciences, Xujiaping 1, Lanzhou, Gansu, 730046, China; b College of Veterinary Medicine, Northeast Agricultural University, Harbin, 150006, China; c Jiangsu Co-Innovation Center for the Prevention and Control of Important Animal Infectious Diseases and Zoonoses, Yangzhou University, Yangzhou, 225009, China

**Keywords:** *Theileria annulata*, Octyl gallate, Drug resistance, Tropical theileriosis

## Abstract

*Theileria annulata*, the causative agent of tropical theileriosis, poses a significant threat to cattle industries, a challenge further intensified by increasing drug resistance. In this study, the food-grade phenolic compound octyl gallate (OG) was evaluated for its antitheilerial activity and identified as a potent and selective inhibitor of *T. annulata*. Comparative screening of gallic acid (GA) derivatives revealed that OG exhibited the strongest activity against *T. annulata*-transformed cell lines, with IC_50_ values of 374.0 nM in TaNM cells and 448.7 nM in the buparvaquone-resistant TaXJS cells, while maintaining more than 90% viability in bovine peripheral blood mononuclear cells (PBMCs) at concentrations up to 50 μM, indicating a favorable selectivity profile. At the molecular and cellular levels, OG treatment led to significant downregulation of the parasite genes *TaSP* and *Tap104* and led to the disintegration of the annulate lamellae (AL), a parasite-induced, host-derived structure implicated in host cell manipulation, together with a concentration-dependent induction of apoptosis in TaNM and TaXJS cells. Collectively, these results identify OG as a promising lead compound with activity against *T. annulata*, combining direct effects on the parasite with apoptosis-associated effects in infected host cells. This study provides experimental support for further investigation of OG as a potential therapeutic option for the control of tropical theileriosis, particularly in the context of emerging drug resistance.

## Highlights

• Octyl gallate inhibits drug-sensitive and resistant *Theileria annulata.*
• The compound is active at nanomolar concentrations.
• Octyl gallate disrupts parasite-induced annulate lamellae.
• Treatment triggers apoptosis in infected host cells.

## Graphical abstract

**Figure:**
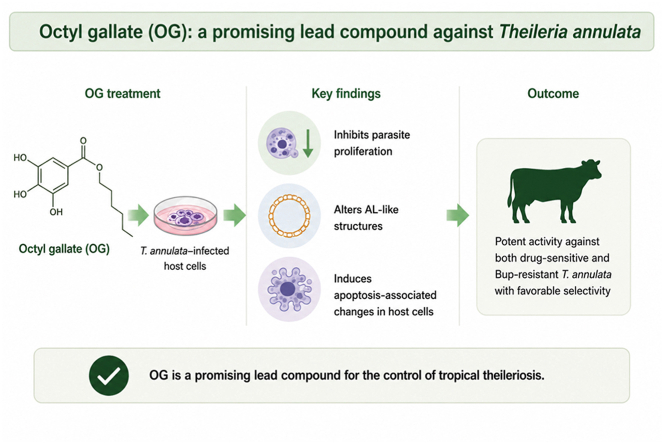

## 1 Introduction

Tropical theileriosis caused by *Theileria annulata* remains one of the most important protozoan diseases affecting cattle in many subtropical and tropical regions (Liu et al., 2022). The parasite, transmitted by ticks, establishes a unique intracellular niche with bovine leukocytes, where it drives host cell proliferation and immune dysregulation (Forsyth et al., 1999; Dobbelaere and Rottenberg, 2003). Infection is characterized by persistent fever, lymphadenopathy, anemia, and marked losses in productivity. Despite long-standing control efforts, *T. annulata* remains a persistent threat to the livestock industry in affected areas (Luo and Lu, 1997; Li et al., 2016).

Control of *T. annulata* infections relies largely on a small number of antiprotozoal compounds, among which buparvaquone (Bup) has been used extensively as the primary therapeutic agent (Hashemi-Fesharki, 1991). While this drug has historically shown high efficacy, accumulating evidence from endemic regions indicates a gradual decline in treatment success. Previous studies have shown reduced sensitivity of *T. annulata* isolates to Bup has been linked to mutations in the parasite *Tapin1* and *Cytb* genes (Ali et al., 2022; Rashid et al., 2024). Nevertheless, the limited availability of alternative drugs means that the development of drug resistance poses a serious threat to the prevention and control of tropical theileriosis.

The emergence of drug resistance has renewed interest in identifying compounds with mechanisms distinct from those of existing antitheilerial agents. In this regard, natural and semi-synthetic phenolic compounds have attracted attention due to their capacity to target essential metabolic pathways in protozoan parasites (Stasiuk and Kozubek, 2010). Octyl gallate (OG), a derivative of gallic acid (GA), is commonly used as an antioxidant in food and pharmaceutical applications, yet its biological activity extends beyond redox scavenging (Qiu et al., 2022). Previous studies have demonstrated inhibitory effects of OG against several protozoan species, with proposed modes of action involving oxidative stress induction and impairment of mitochondrial integrity (Lu et al., 2022). Given the reliance of *T. annulata* on mitochondrial pathways, this compound warrants investigation as a potential antitheilerial agent.

Here, we examine the activity of OG against *T. annulata* schizonts and evaluate its antiparasitic effects. Through this study, we aimed to expand the range of candidate compounds available for the management of tropical theileriosis under conditions of emerging drug resistance.

## 2 Materials and methods

### 2.1 Cell cultures and drug treatments

TaNM (Bup susceptible strain) and TaXJS (a previously characterized Bup resistant strain) were two lines of *T. annulata* schizont-infected bovine lymphocyte (Che et al., 2025). PBMCs were isolated from whole blood by density gradient centrifugation and washed twice with PBS after collection. All cells were cultured in RPMI 1640 medium, supplemented with 10% fetal bovine serum (FBS) at 37°C with 5% CO_2_.

### 2.2 Chemicals

The compounds buparvaquone (Bup), Octyl gallate (OG), gallic acid (GA), Methyl gallate (MG), Ethyl gallate (EG), Propyl gallate (PG), (−)-Epicatechin gallate (-EG), Dodecyl gallate (DG), Stearyl gallate (SG) and Hexyl gallate (HG) were purchased from MedChemExpress (MCE) and stored as a 10 mM stock solution in DMSO (Macklin). Bup (600 nM) and 0.1% DMSO were used as positive control (PC) and negative control (NC), respectively.

### 2.3 Cell viability and proliferation assay in vitro

TaNM cells were plated in 96-well plate with 1.5 × 10^4^ cells per well in 100 μL RPMI 1640 and incubated at 37°C with 5% CO_2_. Different concentrations (10, 7.5, 5, 2.5, 1, 0.75, 0.5, 0.25, 0.1, 0.075, 0.05, 0.025 μM) of compounds were added to wells to incubate for 48 h. Each treatment was tested in triplicate in three independent experiments. Subsequently, 10 μL of Cell Counting Kit-8 (CCK-8; Abbkine Scientific Co., Ltd) was added to each well, followed by a 4 - hour incubation. Cell viability was calculated based on the absorbance measured at 450 nm using a microplate reader.

### 2.4 Cytotoxicity assays with bovine PBMCs

PBMCs from healthy cattle were isolated for evaluation of cytotoxicity, with each treatment tested in triplicate in three independent experiments. A total of 1.5 × 10^4^ cells per well were plated in 96-well plate and treatment with different concentrations (10, 15, 20, 25, 30, 35, 40, 45, 50 μM) of OG. After 48 h, calculated the cell viability using the method described previously.

### 2.5 Parasitological examination

Untreated and OG-treated TaNM and TaXJS cells were fixed on the microscope slide with methanol for 10 min (twice), stained with 5% Giemsa's staining solution for 20 min, rinse the microscope slide with dd H_2_O. Finally, examined under the microscope (Nikon, Japan) using an oil immersion lens at 1000× magnification, and count the number of schizont nuclei within 1000 cells.

### 2.6 RT-qPCR

Total RNA was extracted from untreated and OG-treated TaNM and TaXJS cells from three independent experiments using the RNeasy Mini Kit (Qiagen, Germany) according to the manufacturer's protocol. One microgram of the obtained RNA was then reverse transcribed into cDNA using the PrimeScript™ RT Reagent Kit (Takara). Subsequently, RT-qPCR was performed to analyze relative gene expression levels using TB Green Premix Ex Taq (Takara) on a QuantStudio 5 instrument (Applied Biosystems, USA), following the manufacturer's instructions. The primers used are listed in Supplement 1.

### 2.7 Transmission electron microscopy (TEM)

For TEM, 10^6^ TaNM cells were plated into T25 tissue flasks and cultured for 12 h. Then, the cultures were exposed to 0.5 μM OG and 0.1% DMSO for 24 h. Cells were harvested primarily fixed with 2.5% glutaraldehyde in 100 mM phosphate buffer (pH 7.4) at 4°C for 4 h. After washing with phosphate buffer, the samples were post-fixed with 1% osmium tetroxide for 1 h. Subsequently, the samples were dehydrated through a graded ethanol series, transitioned with propylene oxide, and infiltrated with a mixture of propylene oxide/epoxy resin followed by pure epoxy resin. The samples were then embedded in Embed-812 resin and polymerized at 60°C for 48 h. Ultrathin sections (60 nm) were cut using an ultramicrotome, double-stained with uranyl acetate and lead citrate. Finally, samples from three independent experiments were examined using a transmission electron microscope (Hitachi HT7700) at 80 kV, and representative images were collected and analyzed.

### 2.8 Cell death and apoptosis assay

Briefly, TaNM and TaXJS cells were treated with OG (0.5 and 1 μM) for 48 h and collected from three independent experiments. Cells (1 × 10^5^) were harvested, washed, and resuspended in 195 μL Annexin V-FITC binding buffer. Subsequently, 5 μL Annexin V-FITC and 10 μL propidium iodide (PI) were added, followed by incubation for 15 min at room temperature in the dark. Samples were then placed on ice and immediately analyzed using a CytoFlex LX flow cytometer (Beckman Coulter, USA). At least 10,000 events were collected for each sample.

### 2.9 Western blot analysis

Untreated and OG-treated TaNM and TaXJS cells from three independent experiments were collected and lysed in RIPA buffer containing protease inhibitors. Total protein concentrations were determined using a BCA protein assay kit. Equal amounts of protein (20 μg) were mixed with loading buffer, denatured at 95°C for 5 min, separated by 10% SDS-PAGE, and transferred onto PVDF membranes. Membranes were blocked with 5% non-fat milk in TBST for 1 h at room temperature and then incubated overnight at 4°C with primary antibodies against cleaved caspase-3 (CST 9661), total caspase-3 (CST 9662), BAX (CST2772), Bcl-2 (CST3498) (all 1:1500), and β-actin (CST 4967, 1:6000). After washing with TBST, membranes were incubated with secondary antibodies against mouse and rabbit (1:2000) for 1 h at room temperature. Protein bands were detected using enhanced chemiluminescence reagent and visualized with a Chemiluminescence Imager (Bio-Rad, USA).

### 2.10 Statistical analysis

All statistical tests were conducted using Prism version 9.0. IC_50_ values were determined by nonlinear regression (curve fitting) analysis. Comparisons among multiple groups were analyzed using two-way ANOVA followed by Dunnett's multiple comparisons test. In the figure, ∗ represents *P* < 0.05; ∗∗ represents *P* < 0.01; ∗∗∗ represents *P* < 0.001; ∗∗∗∗ represents *P* < 0.0001 by ANOVA.

## 3 Results

### 3.1 Screening identified OG as an active compound

**Fig. 1:**
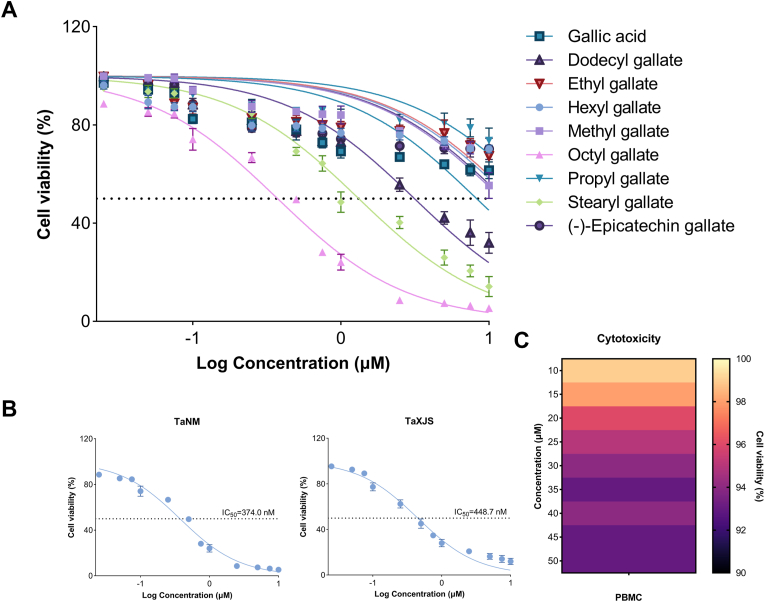
Effect of compounds on cells. A. The graph illustrates the reduction in growth of TaNM cells incubated with different doses of 9 compounds. B. The IC_50_ values of OG for TaNM and TaXJS cells. C. PBMCs isolated from healthy cattle were used to evaluate the host cells cytotoxicity of OG.

TaNM cells were individually treated with 9 compounds at different concentrations. Cell viability was measured by CCK8 assay and calculated the IC_50_. Three compounds, OG, SG, and DG, exhibited relatively good inhibitory activity against TaNM cells (Fig. 1A, Table 1), and OG showed the best inhibitory effect with an IC_50_ of 374.0 nM (Fig. 1B). The inhibitory effect of OG on TaXJS cells was calculated in the same way, with an IC_50_ of 448.7 nM (Fig. 1B).

**Table 1:** Summary of gallic acid and its derivatives.

Compound	Structure^a^	Molecular Weight (g/mol)	LogP^b^	IC_50_^c^ (μM) TaNM
Gallic acid		170.12	0.91	8.263
Dodecyl gallate		338.4	7.38	3.143
Ethyl gallate		198.17	2.07	14.74
Hexyl gallate		254.28	4.19	13.97
Methyl gallate		184.15	1.54	12.18
Octyl gallate		282.33	5.26	0.3740
Propyl gallate		212.2	2.60	21.46
Stearyl gallate		422.6	10.57	1.321
(−)-Epicatechin gallate		442.37	2.67	12.69

a Structures were derived from MCE website (www.medchemexpress.cn).

b logP: Distribution coefficient octanol/water.

c IC_50_ = the concentration resulting in cytotoxicity in 50% of cultured cells after 48 h of treatment, each treatment was tested in triplicate in three independent experiments.

OG exhibits low cytotoxicity, as supported by both our experimental data and previously reported values. In our study, treatment with 50 μM OG maintained viability above 90% in PBMCs (Fig. 1C), indicating minimal adverse effects on primary cells at this concentration. As the CC_50_ value in PBMCs was estimated to be > 50 μM, the selectivity indices were >133.7 for TaNM cells and >111.4 for TaXJS cells.

### 3.2 OG potently inhibited proliferation of *T. annulata*

**Fig. 2:**
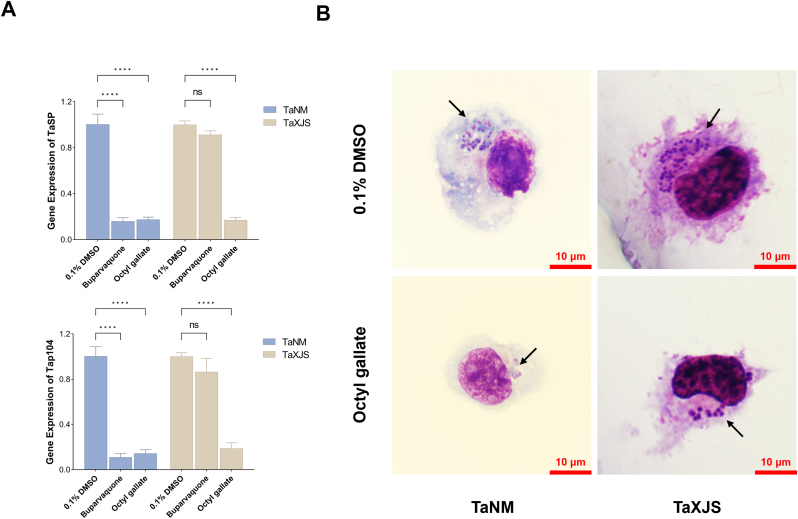
OG inhibits the proliferation of *T. annulata*. A. OG reduces the mRNA levels of TaSP and Tap104. B. OG reduced the number of *T. annulata* schizont nuclei in TaNM and TaXJS cells, the arrows indicate the *T. annulata* schizont nuclei.

We treated TaNM and TaXJS cells with OG (500 nM), Bup (600 nM, PC), and 0.1% DMSO (NC) for 24 h, assessed the mRNA levels of *TaSP* and *Tap104* relative to *T. annulata* *Actin* via RT-qPCR (Hostettler et al., 2014). The primer sequences used for RT-qPCR are provided in Supplement 1. We found that OG suppressed the expression of both genes in both cell lines. However, Bup elicited a differential effect, reducing their expression solely in TaNM cells, with no significant impact in TaXJS cells (Fig. 2A).

TaNM and TaXJS were treated with OG and 0.1% DMSO for 24 h, respectively, followed by Giemsa staining and microscopic examination. The results showed that OG treatment resulted in a reduction in the number of *T. annulata* schizonts within both TaNM and TaXJS cells (Fig. 2B), indicating that OG possesses promising antiparasitic activity and effectively inhibits the proliferation of *T. annulata*.

### 3.3 OG caused ultrastructural damage to *T. annulata* schizonts

**Fig. 3:**
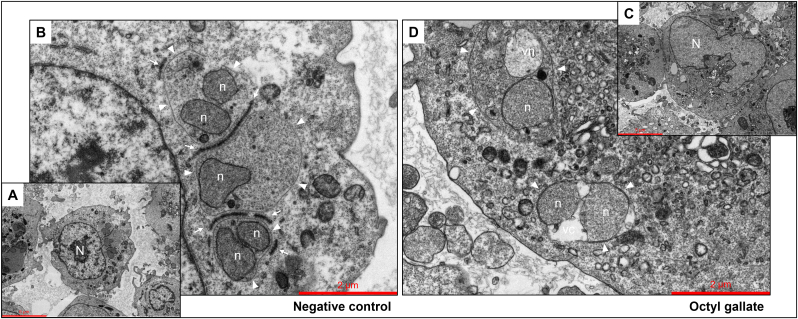
TEM of untreated and OG-treated TaNM cells. A shows untreated TaNM cells (NC), while B presents a higher-magnification view of the boxed area in A. C shows OG-treated TaNM cells, while D presents a higher-magnification view of the boxed area in C. The triangle indicates the schizont-host cell cytoplasm interface; the arrows indicate AL; N: host cell nucleus; n: schizont nuclei; vn: vacuolization within the schizont nuclei; vc: vacuolization within the schizont cytoplasm.

In normally cultured TaNM cells (Fig. 3A and B, with B showing a higher-magnification view of the boxed area in A), the host cell nucleus and multiple schizont nuclei were readily identifiable. Several schizont nuclei were enclosed within a single parasitophorous membrane, demarcating them from the host cell cytoplasm.

Following a 24 h treatment with OG (Fig. 3C and D, with D showing a higher-magnification view of the boxed area in C), a marked reduction or complete disappearance of these annulate lamellae (AL) was noted. Concurrently, the schizont nuclei exhibited apparent swelling, and some showed signs of vacuolization, indicating ultrastructural alterations consistent with parasite injury.

### 3.4 OG promoted apoptosis in TaNM and TaXJS cells

**Fig. 4:**
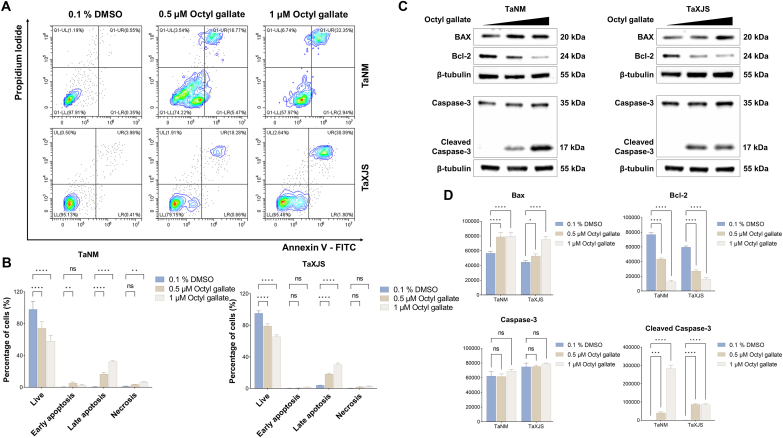
OG induced apoptosis in TaNM and TaXJS cells. A. Representative flow cytometry analysis of 0.1% DMSO and OG (0.5 μM and 1 μM) treated TaNM and TaXJS cells. TaNM and TaXJS cells were stained using Annexin V/PI. B. Quantitative analysis showing the percentages of live, early apoptotic, late apoptotic, and necrotic cell populations. C. Representative Western blot analysis of apoptosis-related proteins. D. Quantitative analysis of Western blot analysis.

To investigate the mode of action by which OG inhibits cell growth, apoptosis was assessed using Annexin V/PI double staining followed by flow cytometry (Fig. 4A). TaNM and TaXJS cells were treated with 0.5 μM or 1 μM for 48 h, then collected and analyzed. The results demonstrated that OG treatment significantly induced apoptosis in both cell lines in concentration-dependent manner. Following exposure to 0.5 μM OG, the viable cell population decreased by 23.69% and 15.98% in TaNM and TaXJS cells, respectively (*P* < 0.0001), while the late apoptotic cells proportion increased by 16.22% and 14.32%, respectively (*P* < 0.0001). At the higher concentration of 1 μM OG, the reduction in viable cells was more pronounced, with declines of 39.94% (TaNM, *P* < 0.0001) and 29.65% (TaXJS, *P* < 0.0001). Concurrently, the late apoptotic cells populations increased notably to 31.8% and 26.13% in TaNM and TaXJS cells, respectively (*P* < 0.0001). These data indicate that OG can promote apoptosis in both TaNM and TaXJS cells.

Western blot analysis was performed to evaluate the effects of OG treatment on apoptosis-related proteins in TaNM and TaXJS cells. OG treatment resulted in a marked increase in the expression of the pro-apoptotic protein BAX in both cell types (Fig. 4C). In contrast, the expression level of the anti-apoptotic protein Bcl-2 was significantly reduced following OG treatment. Consistent with these changes, the level of cleaved caspase-3 was notably elevated in OG-treated cells compared with the control group.

The concurrent upregulation of BAX and downregulation of Bcl-2 was associated with an increased BAX/Bcl-2 ratio in TaNM and TaXJS cells after OG treatment. In addition, the increased level of cleaved caspase-3 further supports the activation of apoptosis-associated signaling.

## 4 Discussion

Tropical theileriosis, caused by *T. annulata*, is one of the major parasitic diseases affecting global livestock production, leading to substantial economic losses annually. Bup remains the first-line therapeutic agent widely used for treatment; however, the emergence of drug resistance poses a serious challenge to disease control (Rashid et al., 2024). GA, a naturally abundant polyphenol especially prevalent in plants, is known to exhibit diverse biological activities including antioxidants, antibacterial, antiviral, and anticancer effects (Verma et al., 2013; Al Zahrani et al., 2020). Its derivative, OG, a common food additive, has been reported to possess broad-spectrum antimicrobial and anticancer properties (Wolf et al., 2017; Sales et al., 2018).

In this study, through screening GA and its derivatives (9 compounds in total, Fig. 1A), we found that OG showed pronounced anti-parasitic activity against the *T. annulata*-transformed cell line TaNM, with an IC_50_ of 374.0 nM (Fig. 1B). This suggests that the octyl sidechain may contribute to enhanced membrane permeability and intracellular activity against the parasite. Notably, OG retained high potency against the Bup-resistant TaXJS cell line (IC_50_ = 448.7 nM). Giemsa-staining and microscopic examinations further revealed that the number of *T. annulata* schizont nuclei in both TaNM and TaXJS cells decreased after OG treatment (Fig. 2B). The qPCR analysis strengthened these findings: OG markedly down-regulated the expression of key parasite genes *TaSP* and *Tap104* in the resistant strain, whereas Bup had minimal effect (Fig. 2A). These results suggest that OG may act through a mechanism distinct from that of Bup, which targets the mitochondrial electron-transport chain (Mhadhbi et al., 2015), and may bypass existing resistance pathways. Thus, OG represents not only an effective inhibitor but also a potential novel chemical scaffold (a core structure for designing derivative compounds) for addressing clinical drug resistance.

To evaluate the safety profile of OG, we isolated PBMCs from healthy cattle and performed cytotoxicity assays. The results showed that treatment with 50 μM OG maintained >90% cell viability (Fig. 1C), indicating minimal toxicity to primary cells at this concentration. This is consistent with literature reports that the IC_50_ of OG against human CCRF-CEM cells and mouse B16F10 cells are 58.5 μM and 45 μM, respectively (Locatelli et al., 2013; Jara et al., 2014). Collectively, these data demonstrate that OG exhibits favorable selectivity across different cell types, with significant cytotoxicity only at concentrations far exceeding those required for anti-parasitic activity. Hence, at the in vitro concentrations effective against *T. annulata*, OG is well tolerated by non-target cells, supporting its potential as a lead compound for further development against *T. annulata.*

AL (Jura et al., 1983; Huber et al., 2020), also known as electron-dense button-like structures (BLS) (Hostettler et al., 2016), were frequently observed surrounding the schizonts. Biochemically and structurally analogous to nuclear pore complexes, they dynamically reorganize in synchrony with the host cell cycle and selectively recruit core components of the host nuclear transport machinery. This forms a critical schizont-host cell interface, leading to the prevailing hypothesis that AL functions as a signaling hub for cell cycle synchronization and a specialized conduit for trafficking parasite effector proteins into the host nucleus to reprogram gene expression (Huber et al., 2020; Woods et al., 2021).

The marked reduction or disassembly of AL following OG treatment, as observed by TEM (Fig. 3), suggests that OG may affect parasite-associated interface structures. By disrupting this specific interface, OG may impair the parasite's ability to sense the host cell cycle and to translocate effector molecules, thereby weakening host cell manipulation mechanisms. These observations may provide a possible structure-based explanation for the anti-parasitic activity of OG and its apoptosis-associated effects in infected cells. However, additional mechanistic studies will be required to further clarify the relationship between AL alterations and the anti-parasitic effects of OG.

In addition to direct parasite suppression, OG effectively induced apoptosis in TaNM and TaXJS cells in a concentration-dependent manner. *Theileria*-infected cells acquire a transformed, immortalized phenotype reminiscent of cancer cells, enabling them to resist apoptosis, which is considered a hallmark of persistent infection (Shiels et al., 2006; Kinnaird et al., 2013). OG successfully reversed this phenotype and partially restored apoptotic responses in infected cells (Fig. 4). This effect may be associated with the disruption of AL, as AL mediated survival signals could be interrupted. Alternatively, the apoptosis-associated changes may reflect broader cellular responses induced by OG treatment. Western blot detection of apoptotic markers provided molecular level corroboration. Inducing apoptosis in infected cells essentially “clears” the parasite-transformed “factories” from the host side, offering strong support for the therapeutic potential of OG in more complex physiological settings.

An intriguing observation from our screening further illuminates the structure activity relationship: while OG (C8 chain) exhibited the highest anti-parasitic activity, its longer-chain homologs, DG (C12) and SG (C18), showed reduced potency in the primary screening using TaNM cells (Table 1). This cannot be explained by logP alone, which increases monotonically with chain length, but rather reflects a classic “lipophilicity window”. The C8 chain of OG appears to reside at an optimal point within this window, providing sufficient membrane permeability for intracellular access while retaining adequate aqueous dispersibility for target engagement within the cytosol. In contrast, the higher logP of the longer chains likely leads to excessive membrane retention or poor cytosolic solubility, diminishing effective target exposure.

While the logP (5.26) underpins OG's excellent in vitro activity, this very property also defines its primary developability challenge: poor aqueous solubility and the risk of off-target distribution. Therefore, future medicinal chemistry efforts should focus on rational structural modifications to modulate the lipophilicity profile (Cao et al., 2022). Given its relatively high lipophilicity, introducing hydrophilic moieties, designing prodrugs, or exploring advanced delivery systems may improve the aqueous solubility and bioavailability of OG while preserving its anti-parasitic activity (Carneiro et al., 2019; Feuser et al., 2019; Cai et al., 2024), ultimately yielding candidate compounds with an optimal balance of potency, selectivity, and developability.

## 5 Conclusion

In summary, our study demonstrates that OG is an effective candidate compound against *T. annulata*. OG showed potent activity against both drug-sensitive and Bup-resistant cell lines, inhibited parasite proliferation, and induced apoptosis-associated changes in infected host cells. TEM observations further suggested alterations of AL structures following OG treatment. Collectively, these findings support the potential of OG as a promising lead compound for the treatment of theileriosis.

## Consent for publication

Not applicable.

## Data statement

All relevant data are within the manuscript.

## Ethics approval

In the present study, all the animal experiments were approved by the Animal Ethics Committee of the Lanzhou Veterinary Research Institute, Chinese Academy of Agricultural Sciences. All experimental animals used were dealt with according to the Animal Ethics Procedures and Guidelines of the People's Republic of China (LVRIAEC-2024-034).

## Funding statement

The study was financially supported by the 10.13039/501100012166National Key Research and Development Program of China (2024YFD1800100), the Innovation Program of 10.13039/501100005196Chinese Academy of Agricultural Sciences (CAAS-ASTIP-2021-LVRI), NBCITS (CARS-37), National Parasitic Resources Center (NPRC-2019-194-30).

## CRediT authorship contribution statement

**Jin Che:** Formal analysis, Writing – original draft. **Yixuan Wu:** Writing – original draft. **Yikang Chen:** Methodology. **Jinming Wang:** Conceptualization. **Qingli Niu:** Resources. **Wei Li:** Resources. **Shuai Yang Zhao:** Writing – review & editing. **Guiquan Guan:** Funding acquisition, Supervision. **Hong Yin:** Funding acquisition, Supervision.

## Competing interest

The authors declare that there is no conflict of interest.
