# Supplementary material for: Activity of octyl gallate against drug-sensitive and buparvaquone-resistant Theileria annulata
Source: Int J Parasitol Drugs Drug Resist. 2026 Jul 9;31:100657. doi: 10.1016/j.ijpddr.2026.100657 (PMC13380485; doi:10.1016/j.ijpddr.2026.100657)
Supplement: Multimedia component 1 [file mmc1.docx]

Supplement 1. Primers used in the present study

| Gene | Primer | Sequence | Reference |
| --- | --- | --- | --- |
| Tap104 | Tap104-F | TCATAGGTCTACAGAACTGGA | Hostettler et al. (2014) |
|  | Tap104-R | TTTAGGTGGTTCTGGACCCT |  |
| TaSP | TaSP-F | AGCAGCCCCTTGTCATGGG |  |
|  | TaSP-R | TAATAGCTTTTGCACGGAGGA |  |
| Actin | Actin-F | GAGACCACCTACAACAGCATCATG |  |
|  | Actin-R | CACCTTGATCTTCATGGTGCTGGG |  |
